# Sex-specific radiomic features of L-[S-methyl-^11^C] methionine PET in patients with newly-diagnosed gliomas in relation to IDH1 predictability

**DOI:** 10.3389/fonc.2023.986788

**Published:** 2023-02-03

**Authors:** Laszlo Papp, Sazan Rasul, Clemens P. Spielvogel, Denis Krajnc, Nina Poetsch, Adelheid Woehrer, Eva-Maria Patronas, Boglarka Ecsedi, Julia Furtner, Markus Mitterhauser, Ivo Rausch, Georg Widhalm, Thomas Beyer, Marcus Hacker, Tatjana Traub-Weidinger

**Affiliations:** ^1^ Center for Medical Physics and Biomedical Engineering, Medical University of Vienna, Vienna, Austria; ^2^ Department of Biomedical Imaging and Image-guided Therapy, Medical University of Vienna, Vienna, Austria; ^3^ Christian Doppler Laboratory for Applied Metabolomics , Medical University of Vienna, Vienna, Austria; ^4^ Clinical Institute of Neurology, Medical University of Vienna, Vienna, Austria; ^5^ Division of Pharmaceutical Technology and Biopharmaceutics, Department of Pharmaceutical Sciences, University of Vienna, Vienna, Austria; ^6^ Ludwig Boltzmann Institute Applied Diagnostics, Medical University of Vienna, Vienna, Austria; ^7^ Clinical University of Neuro-Surgery, Medical University of Vienna, Vienna, Austria

**Keywords:** MET-PET, glioma, sex, IDH1, radiomics, imaging biomarker

## Abstract

**Introduction:**

Amino-acid positron emission tomography (PET) is a validated metabolic imaging approach for the diagnostic work-up of gliomas. This study aimed to evaluate sex-specific radiomic characteristics of L-[S-methyl-^11^Cmethionine (MET)-PET images of glioma patients in consideration of the prognostically relevant biomarker isocitrate dehydrogenase (IDH) mutation status.

**Methods:**

MET-PET of 35 astrocytic gliomas (13 females, mean age 41 ± 13 yrs. and 22 males, mean age 46 ± 17 yrs.) and known IDH mutation status were included. All patients underwent radiomic analysis following imaging biomarker standardization initiative (IBSI)-conform guidelines both from standardized uptake value (SUV) and tumor-to-background ratio (TBR) PET values. Aligned Monte Carlo (MC) 100-fold split was utilized for SUV and TBR dataset pairs for both sex and IDH-specific analysis. Borderline and outlier scores were calculated for both sex and IDH-specific MC folds. Feature ranking was performed by R-squared ranking and Mann-Whitney U-test together with Bonferroni correction. Correlation of SUV and TBR radiomics in relation to IDH mutational status in male and female patients were also investigated.

**Results:**

There were no significant features in either SUV or TBR radiomics to distinguish female and male patients. In contrast, intensity histogram coefficient of variation (ih.cov) and intensity skewness (stat.skew) were identified as significant to predict IDH +/-. In addition, IDH+ females had significant ih.cov deviation (0.031) and mean stat.skew (-0.327) differences compared to IDH+ male patients (0.068 and -0.123, respectively) with two-times higher standard deviations of the normal brain background MET uptake as well.

**Discussion:**

We demonstrated that female and male glioma patients have significantly different radiomic profiles in MET PET imaging data. Future IDH prediction models shall not be built on mixed female-male cohorts, but shall rely on sex-specific cohorts and radiomic imaging biomarkers.

## Background

Gliomas represent approximately a quarter of all primary brain and other central nervous system (CNS) tumors. Approximately 81% of malignant tumors cause mortality and morbidity that is disproportionate to their relatively rare incidence (1). To establish a diagnosis with prognostic estimation and an appropriate treatment strategy, histological and molecular feature examination of the glioma tissue as well as contrast-enhanced magnetic resonance imaging (MRI) - the primary imaging modality in brain tumors - are essential. Molecular imaging, using positron emission tomography (PET), is increasingly utilized to compliment MRI in the clinical management of glioma. Radiolabeled amino-acids, such L-[S-methyl-^11^C]methionine (MET), O-(2-[^18^F]-fluoroethyl)-L-tyrosine (FET) or 3,4-dihydroxy-6-[^18^F]-fluoro-l-phenylalanine (FDOPA) are well accepted as a highly-sensitive tracers for glioma characterization prior to treatment planning (2).

Since the introduction of the World Health Organization (WHO) 2016 classification, gliomas have been categorized into molecular subgroups with varied molecular markers and clinical behavior (3). With the 5th edition (2021), the role of molecular diagnostics is emphasized even more strongly. For example, isocitrate dehydrogenase (IDH) mutant astrocytomas are graded 2, 3 or 4 based on histological and molecular features (4). In contrast, IDH-wildtype astrocytoma is considered grade 4 glioblastoma GBM), even in cases without necrosis or vascular proliferations, as long as further genetic features, such as EGFR amplification, TERT promoter mutation or the combined gain of chromosome 7 and loss of chromosome 10 [+7/-10 are present (4). Hence, the IDH mutation plays a key role for the diagnostic assessments and prognostic rating. Biologically, this mutation leads to an overproduction of the oncometabolite R(−)-2-hydroxyglutarate (5), inducing epigenetic and metabolic reprogramming (6). Furthermore, IDH mutation is associated with a distinct angiogenesis transcriptome signature, that can be predicted non-invasively with MRI-based biomarkers (7).

Sex dysmorphism has a great impact on the incidence, distribution, therapy response, and prognosis of all kinds of cancer independent of race, age, and presence of co-morbidities (8), and has been previously described in CNS tumors (9, 10). Epidemiological studies revealed that gliomas are predominant in male compared to female patients, irrespective of the tumor grade (11, 12). Moreover, male sex is an independent risk factor for a shorter survival among patients with GBM (13). Indeed, sex disparity in the incidence and outcome of human diseases and especially in gliomas are broadly recognized, although in most cases it is not sufficiently understood. Initial explanations for sex-specific differences have been observed in preclinical as well clinical studies investigating GBM, the most malignant and aggressive histologic glioma type (14, 15).

Recently, we have described a retrospectively evaluated cohort of treatment naïve patients of different glioma histology using MET-PET, and demonstrated that amino acid PET has an independent impact on survival outcome (16). Moreover, the value of computer-supported predictive models to envisage survival in amino acid avid, treatment- naïve glioma patients based on PET was demonstrated (17). To date, several studies have investigated the feasibility of tumor characterization with PET, particularly built on radiomic analysis and machine learning (ML) (18). Nevertheless, sex specific aspects of glioma with respect to PET imaging characteristics as an expression for amino acid tumor metabolism activity have not been widely investigated. While significant differences of normal brain uptake in male and female glioma patients have been described (19), the effect of normalizing standardized uptake values (SUV) to tumor-to-background ratio (TBR) values in radiomic models predicting IDH1 mutation status is unknown.

In light of the above, we hypothesize that sex-specific differences have an effect on the performance of radiomic prediction models predicting IDH1 mutation status, which may imply that mixed male-female radiomic prediction models may be prone to underperform in either of the sexes, thus, challenging clinical adoption of such models.

In order to exploit radiomic differences between male and female glioma patients that may influence ML prediction model performances, this study aimed to analyze the distribution of radiomics features in between IDH1+/- female and male cases without building or promoting any ML prediction model. Nevertheless, to investigate the above potential radiomic differences, this study performed data preprocessing and feature ranking steps based on methods routinely performed prior to building ML prediction models. Therefore, this study defined the following objectives: (a) to identify sex-specific SUV and TBR radiomic features in order to understand the underlying imaging characteristics of gliomas in male and female patients, (b) to identify potential SUV and TBR radiomic features that are significant to differentiate IDH+ and IDH- patients, and (c) to compare the identified high-ranking radiomic features to differentiate IDH+ and IDH- cases in female and male patients.

## Patients and methods

### Study population

**Figure 1:**
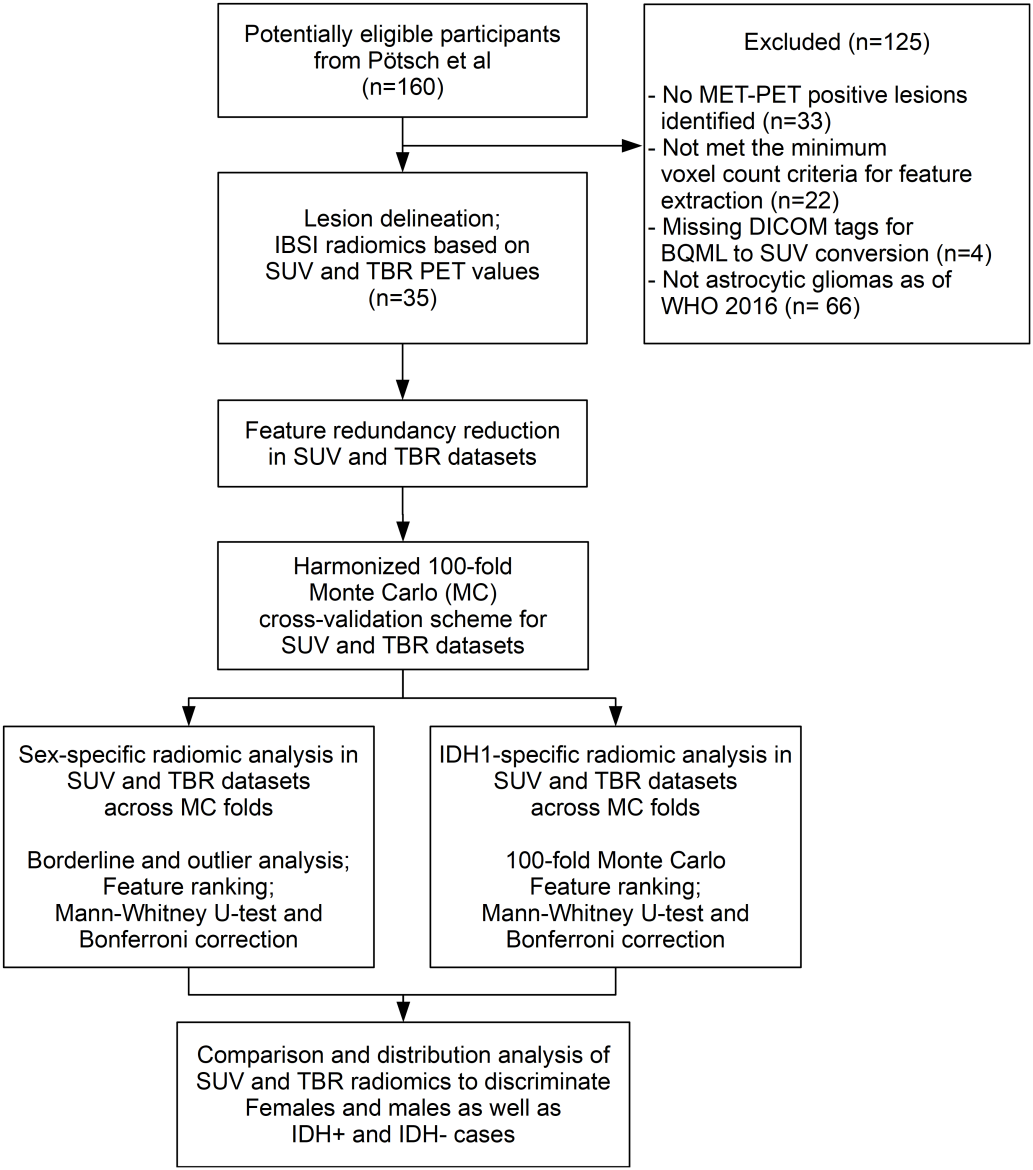
The CONSORT diagram of our study. The Methionine (MET) positive Positron Emission Tomography (PET) cases that met the inclusion criterion underwent lesion delineation and radiomics feature extraction as of the Imaging Biomarker Standardization Initiative (IBSI) guidelines in both standardized uptake value (SUV) and tumor-to-background ratio (TBR) PET configurations. Feature redundancy reduction was followed by aligned 100-fold Monte Carlo (MC) split of SUV and TBR dataset pairs for sex and IDH+/- labels independently. Borderline and outlier scores followed by identifying sex-specific high-ranking radiomic features were done in both SUV and TBR radiomic datasets. The same analysis was performed in parallel to differentiate IDH1+/- cases. Comparison of high-ranking features as well as feature distribution analysis was performed independently in IDH+ and IDH- male and female cases to understand if IDH predictability is associated with sex-specific radiomic patterns in MET-PET.

Approved by the local ethics committee (No 1429/2016), L-[S-methyl-^11^Cmethionine (MET) PET data representing MET-avid gliomas, having IDH1-R132H mutation status and a minimum PET voxel count for radiomic analysis (see **Supplemental Section I**) were selected from a recently published study database by Poetsch et al. (16) (**Figure 1**). This study included astrocytic gliomas resulting in a collection of 35 patients aged above 18 years characterized according to the WHO classification 2016. The IDH1-R132H (IDH) mutation status was determined by immunohistochemistry in all patients, and confirmed by DNA sequencing in patients under 55 years with negative immunohistochemical result. For further evaluation astrocytic tumors were categorized according their IDH mutation status and patient sex. Clinical characteristics of these patients are presented in **Table 1**. All patients underwent a MET-PET imaging at the time of glioma diagnosis prior to any therapy.

**Table 1:** Demographic and MET-PET imaging characteristics of the study population of 35 astrocytic gliomas based on the WHO classification 2016 (n=35).

Parameters	Female (n: 13)	Male (n: 22)
**Age in years (mean ± SD)**	41 ± 13	46 ± 17
IDH1-R132H positive	6	8
IDH1-R132H negative	7	14
**Astrocytoma and GBM (n)**	13	22
Grade 2	6	7
Grade 3	6	13
GBM	1	2
**Tumor SUV (mean ± SD)**	1.51 ± 0.57	1.38 ± 0.28
IDH1-R132H positive	1.20 ± 0.26	1.40 ± 0.25
IDH1-R132H negative	1.78 ± 0.64	1.38 ± 0.31
**CBA SUV (mean ± SD)**	1.14 ± 0.25	1.07 ± 0.17
IDH1-R132H positive	1.19 ± 0.31	1.15 ± 0.16
IDH1-R132H negative	1.11 ± 0.16	1.03 ± 0.16

MET, Methionine; IDH1-R132H, Isocitrate dehydrogenase 1; GBM, glioblastoma, SD, Standard deviation; CBA, cerebral background activity, SUV, standardized uptake value.

### MET-PET imaging

Twenty minutes after an intravenous injection of about 740 MBq of in-house produced MET, a PET examination using a GE Advanced PET system (General Electric Medical System) with a 10-min emission and a 5-min transmission scan for attenuation correction was acquired (16). Image reconstruction was done using filtered back projection with a Hanning filter (cutoff value = 6.2mm), resulting in 35 image slices (4.25 mm slice thickness) with a matrix size of 128 x 128. After reconstruction, an additional 5mm Gaussian post-filtering was applied to the images.

### Lesion delineation and radiomic feature extraction

Delineations of the primary tumors and the corresponding background reference region in MET-PET examinations were performed with the Hermes Hybrid 3D software (Hermes Medical Solutions, Stockholm, Sweden) by three-dimensional iso-count semi-automated tools. The delineated lesions of the 35 cases were validated and analyzed by two nuclear medicine physicians. In all patients, the SUV of each lesion were also normalized to the SUV mean of the respective non-tumoral contralateral background reference region to obtain TBR values (17). Extraction of 154 radiomic features was performed from the SUV and TBR-normalized amino acid avid lesions in compliance with the Imaging Biomarker Standardization Initiative (IBSI) (20). See **Supplemental Table 1** for details of the IBSI-conform feature extraction.

### Data preprocessing

Since many radiomic features represent a generally high redundancy, the extracted 154 features of both SUV and TBR datasets underwent correlation matrix analyses with an absolute Pearson correlation coefficient threshold of 0.85 to identify redundant feature clusters (21). The feature with the highest variance was selected from each redundant cluster for further analysis in both SUV and TBR datasets. Since this step only analyzed radiomic feature pairs without relying on the patient sex or the IHD1 reference standards, the redundancy reduced SUV and TBR sets were used for all subsequent analyses of this study.

### Harmonized cross-validation scheme

Both redundancy-reduced SUV and TBR datasets underwent a 100-fold Monte Carlo (MC) split (21) to simulate a cross-validation scheme within a single-center study. In each fold, one male and one female were randomly selected to serve as held-out set, while the remaining cases were assigned to the given training set of the given fold. The same split configuration was utilized for both SUV and TBR datasets. No repetition of fold configurations was allowed. The SUV and TBR datasets also underwent an aligned 100-fold MC CV split as of the IDH+/- status of the patients with leaving out one IDH+ and one IDH- case per fold.

### Borderline and outlier score analyses

Both SUV and TBR datasets underwent borderline and outlier score analyses in the sex and IDH-specific MC CV folds. Borderline scores were calculated across the 100-fold subsets by Tomek links (22). Similarly, for each fold subset, outlier scores were calculated by the isolation forest approach (23). The distribution of borderline-outlier score pairs across MC folds were compared between the SUV and TBR radiomic datasets in the sex and IDH-specific MC CV splits.

### Deciphering sex-specific radiomic differences

Identifying sex-specific radiomic imaging patterns and comparison of them in SUV and TBR datasets relied on feature ranking. To minimize the chances of false discoveries, this study relied a two-step feature ranking process. First, each training subset across the pre-generated Monte Carlo (MC) splits underwent a per-feature R-squared ranking (24) in relation to patient sex. Only the highest-ranking six features that separated best male and female patients were selected as relevant per MC fold. This number was chosen following the “curse of dimensionality” rule in relation to training sample count (24). The Monte Carlo feature ranking in both SUV and TBR datasets was determined by calculating how many times each of the radiomic features occurred across the 100 MC folds. Features with MC occurrence rates higher than 90% were subject to a Mann-Whitney U-test (25). This step was followed by Bonferroni correction with factor of 31 (highest non-redundant feature count), thus, resulting in *p*<0.0016 as significance level (26). This significance level was applied to identify sex-specific features in both SUV and TBR datasets.

### Investigation of IDH+/- predictive performances between male and female patients

Combined Monte Carlo and Mann-Whitney U feature ranking with Bonferroni correction, as in case of to the sex-specific investigation, were performed in relation to discriminate IDH+/- patients in the SUV and TBR datasets.

## Results

### Clinical and MET-PET image characteristics

In total, 35 data sets (13 females, average age of 41 ± 13y, and 22 males with an average age 46 ± 17y) were selected for radiomics feature extraction and radiomics analysis of MET-avid gliomas. In male patients, the average SUV in tumors was 1.40 and 1.38 in IDH+ and IDH- cases, respectively. In contrast, females had an average SUV of 1.20 and 1.78 in IDH+ and IDH- cases, respectively within tumor lesions. The cerebral background activity (CBA) was higher in females than in males, and it was higher in both of them in case of IDH+ cases. The largest CBA spread was seen in IDH+ females (stdev: 0.31) compared to IDH+ males as well as IDH- patients (stdev: 0.16). See **Table 1** for detailed demographics, clinical and imaging characteristics.

### Data preprocessing

In the SUV dataset, feature redundancy reduction reduced the number of extracted radiomic features from 154 to 31. In the TBR dataset, redundancy reduction resulted in 30 features (see **Supplemental Figure 1**).

### Borderline and outlier score analyses

A lower borderline score distribution in the TBR dataset to separate male and female patients compared to the SUV dataset (mean borderline scores: 0.021 in TBR and 0.038 in SUV) was observed. Similarly, a lower mean outlier score in the TBR dataset compared to SUV was present (mean outlier scores: 0.06 in TBR and 0.07 in SUV).

**Figure 2:**
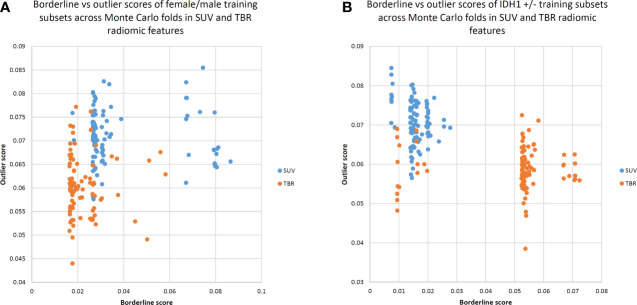
Borderline versus outlier scores of Monte Carlo training subsets for standardized uptake value (SUV) and tumor-to-background ratio (TBR) radiomic features to separate female/male **(A)** as well as IDH1 +/- **(B)** cases. Borderline scores were based on Tomek links (22), while outlier scores were calculated by the Isolation Forest approach (23). TBR features tend to demonstrate lower borderline scores, implying that male and female patients can be better separated in a TBR feature space. Low borderline and outlier scores indicate a better distinction between male and female patients in general.

In contrast, to differentiate IDH+/- cases, TBR datasets demonstrated a much higher borderline range than SUV datasets (mean borderline scores: 0.049 in TBR and 0.015 in SUV). Furthermore, TBR datasets had lower outlier scores compared to SUV datasets (mean outlier scores: 0.058 in TBR and 0.07 in SUV). See **Figure 2** for the borderline-outlier score distributions in the SUV and TBR datasets in relation to sex and IDH+/- differentiations.

### Deciphering sex-specific radiomic differences

**Figure 3:**
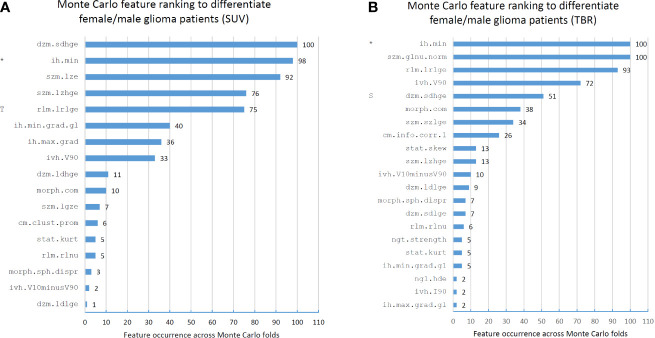
Feature occurrences of the 10 highest-ranking features per Monte Carlo fold that separate male and female patients in standard uptake value (SUV)-based **(A)** and tumor-to-background ratio (TBR)-based **(B)** radiomic datasets. Prefix “T” in the SUV feature ranks imply that the given feature was selected as high-ranking (>90% occurrence) in the TBR dataset. Similarly, prefix “S” in the TBR feature ranks denote a high-ranking SUV feature. Co-occurring high-ranking features in both SUV and TBR datasets are denoted by “*”. Features are denoted by their Imaging Biomarker Standardization Initiative (IBSI) identifiers. For the names of each feature see **Supplemental Table 1**.

The Monte Carlo (MC) feature ranking across all 100 folds resulted in 17 features in the SUV dataset, of which three had an occurrence of >90%. In the TBR dataset, 21 MC features were identified, of which three had an occurrence of >90% (**Figure 3**). The only MC high-ranking feature in both SUV and TBR datasets was the minimum discretized histogram intensity (ih.min). Nevertheless, Mann-Whitney U test revealed no significant features to differentiate patient sex in the either of the datasets (**Table 2**).

**Table 2:** Standardized uptake value (SUV) and tumor-to-background ratio (TBR) value-based glioma radiomic dataset characteristics to differentiate female and male patients.

Dataset	MC-selected features	>90% Occurring features	p-values
SUV	17	ih.min	0.112
		dzm.sdhge	0.147
		szm.lze	0.298
TBR	21	ih.min	0.147
		szm.glnu.norm	0.167
		rlm.lrlge	0.986

MC, Monte Carlo. The high-ranking MC feature list is ordered by ascending p-values in both SUV and TBR datasets independently. Features are denoted by their Imaging Biomarker Standardization Initiative (IBSI) identifiers. For the names of each feature see **Supplemental Table 1**.

### Comparison of IDH+/- predictive performances between male and female patients

**Figure 4:**
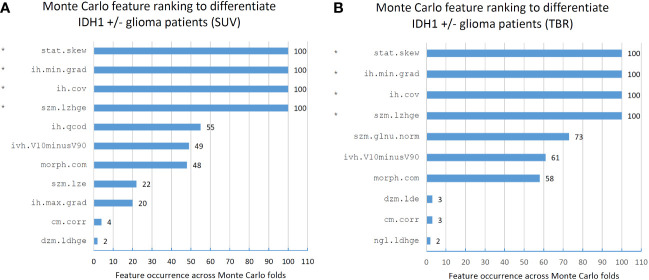
Feature occurrences of the 10 highest-ranking features per Monte Carlo fold that separate IDH1+/- patients in standard uptake value (SUV)-based **(A)** and tumor-to-background ratio (TBR)-based **(B)** radiomic datasets. Co-occurring high-ranking features in both SUV and TBR datasets are denoted by “*”. Features are denoted by their Imaging Biomarker Standardization Initiative (IBSI) identifiers. For the names of each feature see **Supplemental Table 1**.

**Figure 5:**
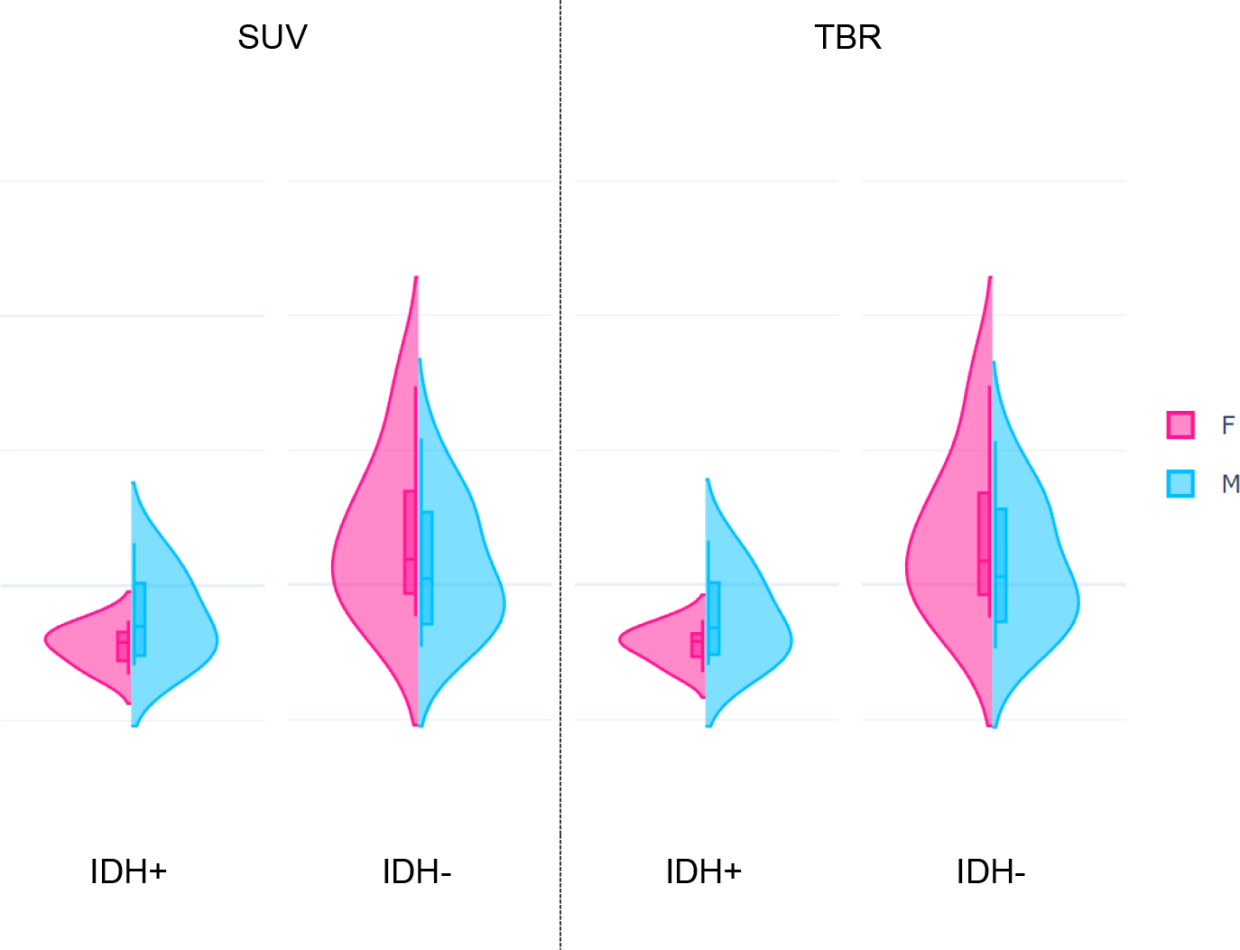
Distributions of the highest-ranking Intensity histogram coefficient of variation (ih.cov) feature extracted from standardized uptake value (SUV, *p*=0.0007) and tumor-to-background ratio (TBR, *p*=0.0003) values of glioma patients. Each plot represents distributions of the feature in female (F) and male (M) patients grouped by the IDH+ (low risk) and IDH- (high risk) mutation statuses.

Feature ranking to discriminate IDH+/- cases resulted in 11 and 10 MC features in SUV and TBR datasets, respectively (**Figure 4**). Of these features, four had a >90% occurrence in both SUV and TBR cases and they were also mutually present in both datasets as MC high-ranking. Mann-Whitney U-test identified the intensity histogram coefficient of variation (ih.cov) as high-ranking in both the SUV (*p*=0.0007) and the TBR (*p*=0.0003) datasets (**Figure 5**). In addition, intensity skewness (stat.skew) was also identified to be significant with *p*=0.0013 in both SUV and TBR datasets (**Table 3**).

**Table 3:** Standardized uptake value (SUV) and tumor-to-background ratio (TBR) value-based glioma radiomic dataset characteristics to differentiate IDH+ and IDH- cases.

Dataset	MC-selected features	>90% Occurring features	p-values
SUV	11	ih.cov	0.0007
		stat.skew	0.0013
		szm.lzhge	0.0044
		ih.min.grad	0.0549
TBR	11	ih.cov	0.0003
		stat.skew	0.0013
		szm.lzhge	0.0028
		ih.min.grad	0.0139

MC, Monte Carlo. The high-ranking MC feature list is ordered by ascending p-values in both SUV and TBR datasets independently. Features are denoted by their Imaging Biomarker Standardization Initiative (IBSI) identifiers. For the names of each feature see **Supplemental Table 1**.

While IDH- female and male patients had similar ih.cov values, IDH+ patients demonstrated a more diverse ih.cov distribution (**Table 4**). As such, IDH+ females had approximately half the ih.cov deviation compared to IDH+ males. Stat.skew also represented similar value distributions in IDH- female and male patients. In contrast, IDH+ females had lower skewness (mean: -0.327) compared to IDH+ males (mean: -0.123) (**Table 4**). **Table 4** includes the distribution of ih.cov and stat.skew features in IDH+ and IDH- female and male patients.

**Table 4:** Mean and standard deviation of histogram coefficient of variation (ih.cov) and intensity skewness (stat.skew) features in IDH+ and IDH- female and male patients in both SUV and TBR datasets.

	ih.cov		stat.skew	
	SUV	TBR	SUV	TBR
Female, IDH-	0.440 ± 0.126	0.438 ± 0.126	0.483 ± 0.683	0.483 ± 0.683
Male, IDH-	0.385 ± 0.100	0.386 ± 0.099	0.519 ± 0.619	0.519 ± 0.619
Female, IDH+	0.264 ± 0.031	0.264 ± 0.029	-0.327 ± 0.562	-0.327 ± 0.562
Male, IDH+	0.308 + 0.068	0.307 ± 0.067	-0.123 ± 0.501	-0.123 ± 0.501

Since stat.skew is a normalized calculation, it is agnostic to TBR normalization, hence, its values are identical in both SUV and TBR datasets.

## Discussion

Sex differences are appreciated as important parameters of human health and disease. Although sex differences in incidence, disease phenotype, and outcome are well described, the molecular bases for sex dimorphism have only recently come to the focus of research, such as in gliomas (27). Moreover, the applications of artificial intelligence (AI) in health care have helped advance the qualitative interpretation of cancer imaging, including volumetric delineation of tumors, extrapolation of the tumor genotype and biological course from its radiographic phenotype, prediction of clinical outcome, and assessment of the impact of disease and treatment (18). Many AI approaches have been created to help with glioma management concentrating on clinical and radiological data from CT and MRI (28). First steps have also been taken with regard to PET as an imaging tool for ML-based analysis of gliomas. Describing the metabolic behavior of the tumor, amino acid PET is particularly under investigation to classify glioma tissue regarding prognosis (29, 30). Considering that sex specific differences could also affect PET phenotypes in gliomas led us to take a closer look at astrocytomas classified according to the IDH mutational status, a leading biomarker of the current and the future WHO CNS classification.

Amino-acid brain tumor PET imaging may be affected in various manners: first, heterogeneous composition of glioma tissue due to different histology or grading based on pathological features, such as edema, microvasculature, cellularity, inflammation, necrosis have been observed by MRI (31, 32). Second, the complex tumor microenvironment, a matrix surrounding tumor cells as a mixture of immune, glia, precursor and endothelial cells and signaling molecules also influences the metabolic behavior of tumors, that can be picked up through radiomics (33). Third, sex disparity in brain metabolism and genome-based sex disparity of the tumor cells have been also described (34). Our results hint at biological sex differences between gliomas at the molecular level using amino-acid PET, which is in line with prior findings (15).

Specifically, our analyses revealed sex-specific amino-acid PET-based radiomic characteristics and their effect on IDH risk prediction, allowing a better understanding of how future prediction models in glioma shall consider sex differences. While no significant differences were identified across SUV or TBR features of MET-PET to differentiate females and males, we identified two significant radiomic features to differentiate IDH low- and high-risk cases. The highest-ranking radiomic feature was Intensity histogram coefficient of variation (ih.cov), which is a simple uptake heterogeneity descriptor, representing the relative standard deviation of uptake occurrences within glioma lesions. Intensity skewness (stat.skew), on the other hand, describes whether the distribution of uptake values is symmetric (~value 0), negative-skewed (frequent high uptakes compared to mean) or positive-skewed (frequent low uptakes compared to mean).

Our borderline and outlier analysis revealed that SUV radiomics increases both outlier and borderline scores to discriminate females and males. This alone may point towards the necessity to normalize SUV values to TBR prior to a radiomics-based analysis to build prediction models for glioma patients. Nevertheless, TBR normalization appears to magnify borderline cases to discriminate IDH1+/- cases. This is in line with our findings regarding standard imaging characteristics in both tumors and CBA, particularly regarding the normal brain region, which is routinely chosen to perform TBR normalization. Here, IDH+ females have shown an average lower and less heterogeneous uptake occurrence pattern compared to IDH+ males. In general, a high amino acid tracer uptake indicates a more aggressive tumor behavior with poor prognosis (35). However, our observations may also be explained by the fact that males had more astrocytomas grade 3 compared to females in our cohort, reflecting a tumor tissue with higher heterogeneity (36).

In addition to the above, IDH+ females had 2-times higher CBA standard deviations compared to all other patient groups. Sexual dimorphism of amino acid metabolism and consequently tumor appearance and prognostic outcome of glioma, androgen receptors are discussed as promotors of tumor progression accompanied by high serum testosterone in male patients with malignant brain tumors (37). Known tumor protective effects of estrogens, particularly during the premenopausal years in females (38), may explain in part our observations. Moreover, sex-specific disparity in immuno-physiological reactions against various cancerous and inflammatory processes are also mediated by the sexual hormones (39). Furthermore, studies also revealed metabolic sex disparity in the healthy brain in terms of cerebral glucose metabolism framing these observations as potential differences in cognitive abilities and emotional processing (40–42). Moreover, Verger et al. demonstrated in a retrospective analysis of patients with suspicious brain lesions and negative amino acid FET brain PET scans an enhanced amino acid metabolism of 23% higher SUVmean values in healthy brain tissues of female compared to male participants (19). These observations point to potential cellular and biological differences that could be related to healthy brain metabolism but also to sex-based disease pathogenesis. Interestingly, we found the most-prominent sex-differences in the IDH+ females compared to both IDH+ and IDH- males. However, we did not find similar results for IDH- females. This may imply that regarding the normal brain metabolism, high-risk gliomas represent sex-independent imaging characteristics. In contrast, low-risk gliomas tend to preserve sex-specific features (27).

Prior works investigated the predictability of IDH mutations in glioma patients relying on MRI and PET. Lohmann et al. (43) classified [18F]FET-PET and PET/MRI cases. They reported 80% and 86% validation accuracies with FET-PET and PET/MRI respectively. Wang et al. (44) reported 0.9 cross-validation AUC to predict IDH mutation built on multi-parametric MRI alone and also identified age as well as sex to be a contributing feature to classify patients. Sakai et al. (45) analyzed glioma patients to build IDH prediction models utilizing MRI radiomics with a cross-validation accuracy of 90%. Cao et al. (46) achieved 0.78 cross-validation AUC to predict IDH mutation status with MRI. Analyzing MET-PET, Zhou et al. (47) classified patients to IDH- vs. wildtype built on SUV analytics with 0.73 AUC. While all studies predicted IDH mutation status with a variable number of cases as well as glioma subtypes, none of them investigated the predictability of their established models in females and males independently and none of them compared SUV and TBR radiomics. In contrast to these studies, our findings point towards the understanding that IDH risk predictability seems to be associated to sex-specific tumor amino-acid uptake characteristics, where high variations are prominently present in IDH+ patients, particularly in females. Consistently, SUV-to-TBR normalization in glioma patients appears advantageous particularly for males, while females demonstrate a more diverse predictive performance pattern in TBR-based IDH models.

This study had limitations of being a single-center study. Furthermore, while prior studies established machine learning prediction models to differentiate IDH+ and IDH- cases, we explicitly focused on the analysis of high-ranking radiomic features by standard methodologies (e.g., feature ranking and selection across a Monte Carlo cross-validation scheme) that are routinely performed prior to building prediction models. While we had a relatively low sample count of our cohort, we wish to reflect on the phenomena that due to the heterogeneous nature as well as imbalance occurrence of glioma subtypes, collecting a large patient cohort suitable for analysis according to the actual WHO classification is generally challenging as also demonstrated by others (30). Nevertheless, we consider our findings representative to demonstrate significant differences across IDH+ and IDH- female and male subgroups, that can influence ML predictive model performances in general. In addition to the above, negative amino acid PET - a feature of lower grade gliomas with better prognosis as compared to amino-acid positive gliomas - were not included, as they could not be subjects of the radiomic analysis.

Our findings have important implications within the field of radiomics, machine learning and brain PET imaging analysis, especially regarding sex disparity on the level of both radiomic feature distributions within tumor lesions as well as in the normal brain. Relevant future research therefore has to reflect on our findings when analyzing glioma patients.

## Conclusions

We demonstrate that significant differences in imaging pattern of MET-PET between female and male astrocytic gliomas that can affect IDH predictability. This is caused mainly by a diverse imaging pattern in IDH+ females. Risk prediction using radiomics analysis of PET data in glioma patients, shall rely on sex-specificity of resulting biomarkers as part of future approaches towards precision medicine.

## Data availability statement

The raw data supporting the conclusions of this article will be made available by the authors, without undue reservation.

## Ethics statement

The studies involving human participants were reviewed and approved by Ethikkomission Medizinische Universität Wien. The patients/participants provided their written informed consent to participate in this study.

## Author contributions

Study design: TT-W, LP, AW, E-MP, JF; Data collection, annotation: NP, SR; Data preprocessing: DK, MM, IR, GW; Data analysis: LP, BE, CS; Supervision and review: TT-W, TB, MH. All authors contributed to the article and approved the submitted version.
