# Supplementary material for: Sex-specific radiomic features of L-[S-methyl-11C] methionine PET in patients with newly-diagnosed gliomas in relation to IDH1 predictability
Source: Front Oncol. 2023 Feb 3;13:986788. doi: 10.3389/fonc.2023.986788 (PMC9936222; doi:10.3389/fonc.2023.986788)
Supplement: Supplementary file 1 [file DataSheet_1.docx]

Sex-Specific Radiomic features of L-[S-methyl-^11^C]methionine PET in Patients with Newly-diagnosed Gliomas in relation to IDH1 predictability

Supplemental

1. **Radiomic Feature Extraction**

**Table 1.** the Imaging Biomarker Standardization Initiative (IBSI) reporting structure of the study. The information presented herein is based on the IBSI guidelines (*1*).

| **Patient** | | |
| --- | --- | --- |
| Volume of Interest | 11C-MET-PET-positive glioma lesions in the brain | |
| Patient Preparation | As of Poetsch et al (*2*) | |
| Radiotracer | ^11^C-MET | L-Smethyl-^11^C-methionine |
| **Acquisition and Reconstruction** | | |
| Protocol | As of Poetsch et al (*2*) | |
| Scanner type | Advance PET system (GE Healthcare) | |
| ^11^C-MET PET | - Static - 1 bed position - 770 ± 106 MBq of ^11^C-MET (mean ± SD; range, 447–972 MBq) - 20 min after injection - 3.125 x 3.125 x 3.125 mm voxel size - 3D FBP, Hamming filter 6.2mm cut-off | |
| **Data conversion** | | |
| Step 1 | BQML voxel units were transformed to weight-normalized SUV automatically by the Hermes Hybrid 3D software. | |
| Step 2 | SUV voxel values transformed to tumor-to-background ratio (TBR) by dividing all voxel values with the mean of the reference region drawn in the contralateral region as a 4x4x4 voxel cuboid VOI in each patient. | |
| **Segmentation** | | |
| Software | Hermes Hybrid 3D ver 4.0.0 | |
| VOI definition | Standard semi-automated iso-count 3D. | |
| Number of experts | 1+1 (1 nuclear medicine expert participated in independent delineations, followed by 1 senior nuclear medicine specialist cross-validation and if necessary, modification of first-round results) | |
| Reference image | PET | |
| **Image / VOI interpolation** | | |
| Method | Kriging interpolation in 3D, including nearest neighbors in distance of voxel size main diagonal (*3*). | |
| Grid | Align by center | |
| Extrapolation beyond original image | Neighbor distance search calculated as original voxel size main diagonal + epsilon. Missing value: image minimum | |
| Voxel dimensions | 2.0 x 2.0 x 2.0 mm | |
| Partially masked voxels (VOI) | Taken if more than half of original voxel area included | |
| **Discretization** | | |
| Method | Fixed bin width, variable number of bins | |
| Bin width | - 0.1 (SUV and TBR datasets) | |
| **Image biomarker computation / Parameters** | | |
| **Intensity features** (21): Minimum, Maximum, Mean, Sum, Variance, Skewness, Kurtosis, Median, 10th intensity percentile, 90th intensity percentile, Interquartile range, Range, Intensity-based mean absolute deviation, Intensity-based robust mean absolute deviation, Intensity-based median absolute deviation, Intensity-based coefficient of variation, Intensity-based quartile coefficient of dispersion, Energy, Root mean square intensity, Local intensity peak, Global intensity peak  **Histogram features** (19): Mean discretised intensity, Discretised intensity variance, Discretised intensity skewness, (Excess) discretised intensity kurtosis, Median discretised intensity, Minimum discretised intensity, Maximum discretised intensity, Intensity histogram mode, Intensity histogram mean absolute deviation, Intensity histogram robust mean absolute deviation, Intensity histogram median absolute deviation, Intensity histogram coefficient of variation, Intensity histogram quartile coefficient of dispersion, Discretised intensity entropy, Discretised intensity uniformity, Maximum histogram gradient, Maximum histogram gradient intensity, Minimum histogram gradient, Minimum histogram gradient intensity  **Intensity histogram features** (6): Volume at intensity fraction 10%, Volume at intensity fraction 90%, Intensity at volume fraction 10%, Intensity at volume fraction 90%, Volume fraction difference between intensity fractions, Intensity fraction difference between volume fractions  **GLCM features** (24): Joint maximum, Joint average, Joint variance, Joint entropy, Difference Average, Difference variance, Difference entropy, Sum average, Sum variance, Sum entropy, Angular second moment, Contrast, Dissimilarity, Inverse difference, Normalised inverse difference, Inverse difference moment, Normalised inverse difference moment, Inverse variance, Correlation, Autocorrelation, Cluster shade, Cluster prominence, Information correlation 1, Information correlation 2  **Morphological features** (16): Volume (mesh), Surface area (mesh), Surface to volume ratio, Compactness 1, Compactness 2, Spherical disproportion, Sphericity, Asphericity, Maximum 3D diameter, Volume density (axis-aligned bounding box), Area density (axis-aligned bounding box), Volume density (convex hull), Area density (convex hull), Moran’s I index, Geary’s C measure, Centre of mass shift  **GLSZM features** (16): Small zone emphasis, Large zone emphasis, Low grey level zone emphasis, High grey level zone emphasis, Small zone low grey level emphasis, Small zone high grey level emphasis, Large zone low grey level emphasis, Large zone high grey level emphasis, Grey level non-uniformity, Normalised grey level non-uniformity, Zone size non-uniformity, Normalised zone size non-uniformity, Zone percentage, Grey level variance, Zone size variance, Zone size entropy  **NGTDM features** (5): Coarseness, Contrast, Busyness, Complexity, Strength  **GLRLM features** (16): Short runs emphasis, Long runs emphasis, Low grey level run emphasis, High grey level run emphasis, Short run low grey level emphasis, Short run high grey level emphasis, Long run low grey level emphasis, Long run high grey level emphasis, Grey level non-uniformity, Normalised grey level non-uniformity, Run length non-uniformity, Normalised run length non-uniformity, Run percentage, Grey level variance, Run length variance, Run entropy  **NGLDM features** (17): Low dependence emphasis, High dependence emphasis, Low grey level count emphasis, High grey level count emphasis, Low dependence low grey level emphasis, Low dependence high grey level emphasis, High dependence low grey level emphasis, High dependence high grey level emphasis, Grey level non-uniformity, Normalised grey level non-uniformity, Dependence count non-uniformity, Normalised dependence count non-uniformity, Dependence count percentage, Grey level variance, Dependence count variance, Dependence count entropy, Dependence count energy  **GLDZM features** (16): Small distance emphasis, Large distance emphasis, Low grey level zone emphasis, High grey level zone emphasis, Small distance low grey level emphasis, Small distance high grey level emphasis, Large distance low grey level emphasis, Large distance high grey level emphasis, Grey level non-uniformity, Normalised grey level non-uniformity, Zone distance non-uniformity, Normalised zone distance non-uniformity, Zone percentage, Grey level variance, Zone distance variance, Zone distance entropy | | |
| Software | MUW radiomics engine ver. 2.0.  Software availability upon reasonable request from the corresponding author. | |
| Distance weighting | No | |
| CM symmetry | Symmetric | |
| CM / ZM distance | Chebyshev distance 1 | |
| CM / ZM aggregation | 3D, full-merging | |
| Exclusion criteria | VOIs with less than 125 voxels were excluded from the analysis | |

1. **Feature redundancy analysis**

| 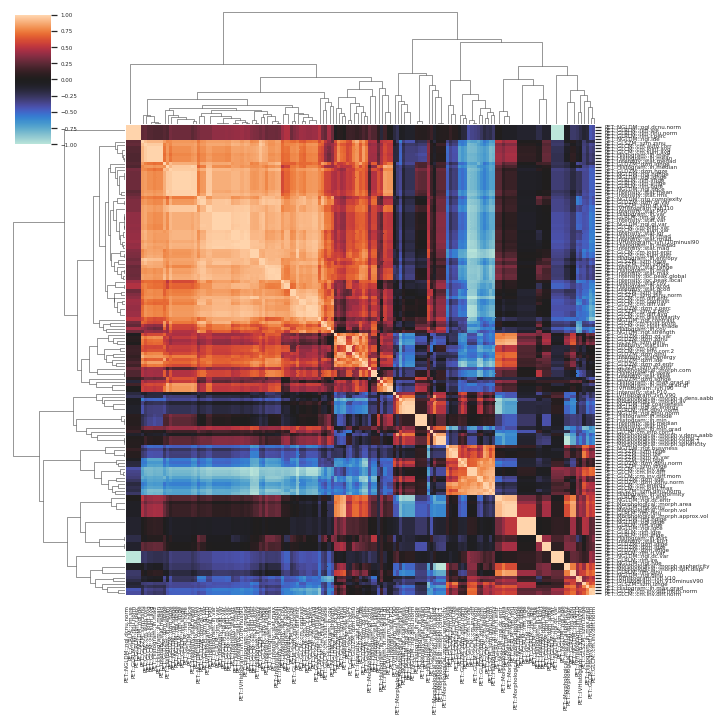 | 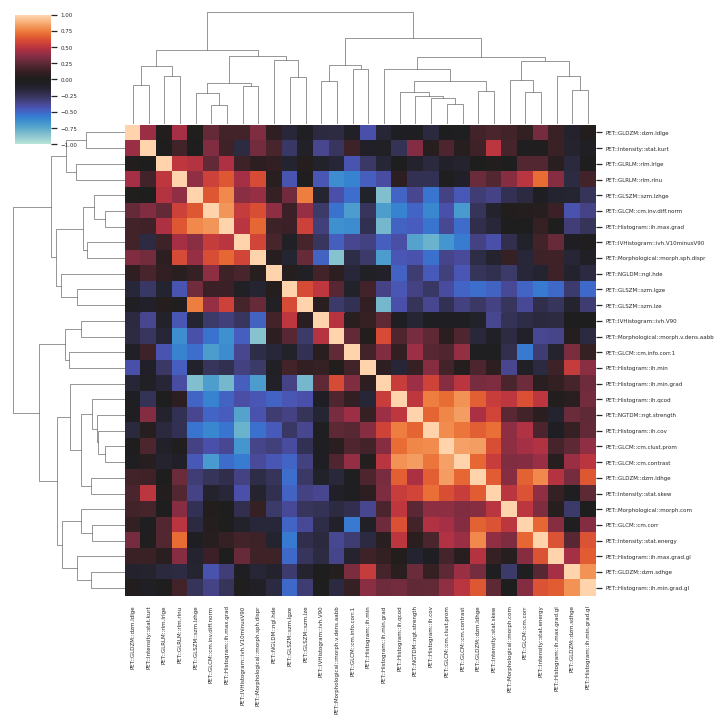 |
| --- | --- |
| (A) | (B) |
| 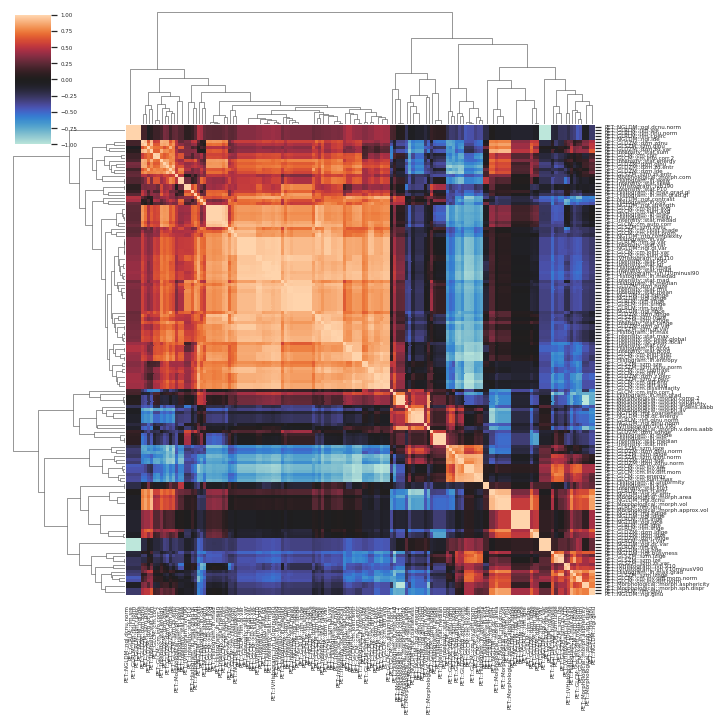 | 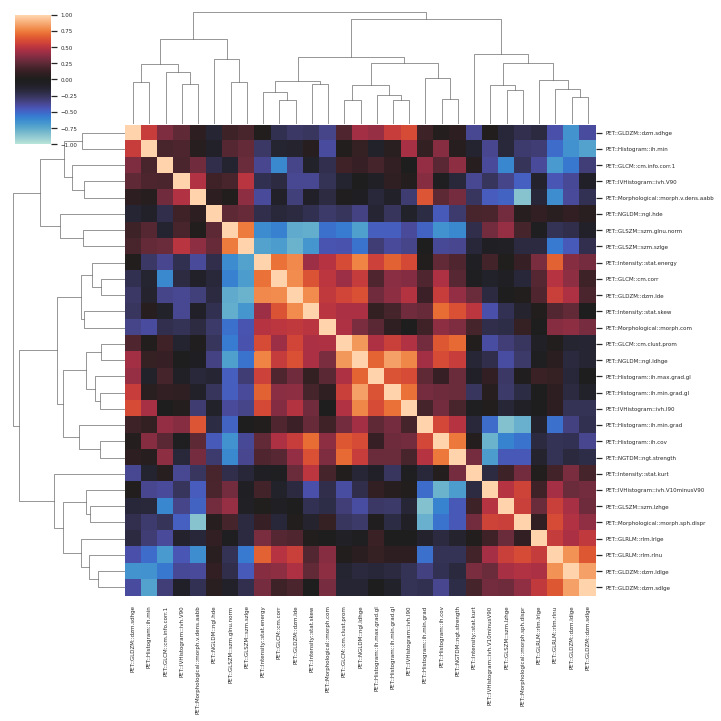 |
| (C) | (D) |

**Figure 1.** Correlation matrices of the radiomic dataset. First row: Standardized uptake value (SUV)-based radiomics redundancy maps before (A) and after (B) redundancy reduction with absolute Pearson correlation coefficient threshold 0.85 Second row: Tumor-to-background ratio (TBR)-based radiomics redundancy maps before (C) and after (D) redundancy reduction with the same parameters.

**References**

1. Zwanenburg A, Leger S, Vallières M, Löck S, Initiative for the IBS. Image biomarker standardisation initiative. *arXiv*. 2016.

2. Poetsch N, Woehrer A, Gesperger J, et al. Visual and semiquantitative11c-methionine pet: an independent prognostic factor for survival of newly diagnosed and treatment-naïve gliomas. *Neuro Oncol*. 2018;20:411-419.

3. Papp L, Poetsch N, Grahovac M, et al. Glioma survival prediction with the combined analysis of in vivo 11c-met-pet, ex vivo and patient features by supervised machine learning. *J Nucl Med*. 2017;59:jnumed.117.202267.
